# Polysialic Acid in the Immune System

**DOI:** 10.3389/fimmu.2021.823637

**Published:** 2022-02-11

**Authors:** Tania M. Villanueva-Cabello, Lya D. Gutiérrez-Valenzuela, Roberta Salinas-Marín, Delia V. López-Guerrero, Iván Martínez-Duncker

**Affiliations:** ^1^ Laboratorio de Glicobiología Humana y Diagnóstico Molecular, Centro de Investigación en Dinámica Celular, Instituto de Investigación en Ciencias Básicas y Aplicadas, Universidad Autónoma del Estado de Morelos, Cuernavaca, Mexico; ^2^ Instituto de Biotecnología, Universidad Nacional Autónoma de México, Cuernavaca, Mexico; ^3^ Facultad de Nutrición, Universidad Autónoma del Estado de Morelos, Cuernavaca, Mexico

**Keywords:** polysialic, glycan, immunity, sialic, glycosylation

## Abstract

Polysialic acid (polySia) is a highly regulated polymer of sialic acid (Sia) with such potent biophysical characteristics that when expressed drastically influences the interaction properties of cells. Although much of what is known of polySia in mammals has been elucidated from the study of its role in the central nervous system (CNS), polySia is also expressed in other tissues, including the immune system where it presents dynamic changes during differentiation, maturation, and activation of different types of immune cells of the innate and adaptive response, being involved in key regulatory mechanisms. At least six polySia protein carriers (CCR7, ESL-1, NCAM, NRP2, ST8Sia 2, and ST8Sia 4) are expressed in different types of immune cells, but there is still much to be explored in regard not only to the regulatory mechanisms that determine their expression and the structure of polySia chains but also to the identification of the *cis*- and *trans*- ligands of polySia that establish signaling networks. This review summarizes the current knowledge on polySia in the immune system, addressing its biosynthesis, its tools for identification and structural characterization, and its functional roles and therapeutic implications.

## Introduction

The immune system is the repertoire of processes contained in the innate and adaptive responses that protect our organism from foreign antigens such as microbes, viruses, cancer cells, and toxins (1). In many of these processes, glycosylation is involved. Glycosylation is the biosynthesis and attachment of carbohydrate structures known as glycans to proteins, lipids, and RNA to form glycoconjugates, albeit they can also be found in a soluble form (2, 3). Glycans participate in different key aspects of the immune system, including the recognition of self and non-self. Additionally, the dynamics and richness of the biological information encoded in glycans and the effector functions of glycan binding proteins allow the fine-tuning and control of the immune response (4, 5).

The diversity of glycans results not only from the variety of linkages that are found between monosaccharides but also from different glycosylation pathways that occur in the endoplasmic reticulum/Golgi apparatus through the coordinated action of glycosyltransferases and glycosidases, as well as other types of proteins involved in supporting their function (nucleotide-sugar synthesis and transport, trafficking, organelle pH, etc.) (6).

A common feature of glycans that stands out is the presence of the negatively charged monosaccharide sialic acid (Sia) in the non-reducing terminus. The fine-tuning of the immune response is highly influenced by the presence/absence and type of linkage of Sia (7). Sialic acids are a family of monosaccharides characterized by a nine-carbon structure with a negative charge in the carboxylate (C1). The sialic acids found in mammalian organisms vary in their substituent at C5, which in *N*-glycolylneuraminic acid (Neu5Gc) is a glycolylated amino group, in *N*-acetylneuraminic acid (Neu5Ac) is an acetylated amino group, and in 2-keto-3-deoxy-nonulosonic acid (Kdn) is a hydroxyl group (7). The glycosidic linkage between the C2 of Sia and the underlying monosaccharide may be α2,3- or α2,6- to galactose (Gal) or *N*-acetylgalactosamine (GalNAc), or α2,8- to another Sia forming chains that vary in length as disialic acid (diSia), oligosialic acid (oligoSia), or polysialic acid (polySia) structures with degrees of polymerization (DP) of 2, 3–7, and 8–400, respectively.

The type of Sia, the chemical modifications it can be subject to including acetyl, sulfonyl, lactyl, methyl, and lactone groups, and the configuration of the glycosidic linkage constitute stereospecific biophysical information that cells can dynamically modify and also be sensed by specific endogenous glycan-binding proteins such as selectins, sialic acid-binding immunoglobulin-type lectins (Siglecs), or CD28, thus establishing key functional pathways in the immune response (8, 9).

In mammals, sialic acids are very abundant, and a single cell displays millions of Sia molecules (7). Sia-containing glycans, including polySia glycans, can work as immune checkpoints for differentiation, maturation, migration, tolerance, and activation, also being involved in the pathogenesis of inflammatory disorders and cancer (7, 10).

Mammalian polySia is characterized as a long polymer (8–400) of terminal α2,8-linked Sia that can be found on *N*-linked and *O*-linked glycans of a restricted group of glycoproteins. PolySia presents with a characteristic enormous hydrated volume and negative charge that strongly modulates the repulsion/attraction between cells (7, 11). Three polysialyltransferases (polySTs), ST8Sia 2, ST8Sia 3, and ST8Sia 4 with distinct tissue expression patterns are involved in the synthesis of polySia in the Golgi, using CMP-Sia as donor substrate (12). The ST8Sia 3 is considered a polyST on the basis that it is capable of autopolysialylation; however, other natural acceptors for polysialylation are not known (13, 14).

Aside from autopolysialylation of ST8Sia 2, ST8Sia 3, and ST8Sia 4 (13, 15, 16), eight other polySia protein carriers have been identified in mammals: chemokine receptor CCR7, CD36, E-selectin ligand1 (ESL-1), neural cell adhesion molecule (NCAM), neuropilin-2 (NRP2), megalin, skeletal muscle α-subunit of the voltage-gated sodium channel, and SynCAM 1 (17–24, 26) (**Table 1**). Of these, NCAM is by far the most studied and characterized and much of what is known on polySia has been obtained from understanding the role of polySia-NCAM in the central nervous system (CNS) (75). Nonetheless, polySia has been also identified in the immune system and can be expressed by different types of cancer cells (44, 76–78). As will be addressed in this review, polysialylated proteins expressed by different immune cells include CCR7, ESL-1, NCAM, NRP2, ST8Sia 2, and ST8Sia 4, although data indicate that other protein carriers remain to be identified.

**Table 1:** Mammalian polysialylated proteins.

Protein	Molecular size	PolySia glycan	PolyST	Function	Immune cell expression
CCR7	378 aa	*N*- and *O*-glycans (25)	ST8Sia 4 (25)	Lymphocyte and DC homing to the lymph nodes and intestinal Peyer’s patches (27, 28)	Activated B cells, naive T cells, regulatory and memory T cells, NK cells, and DCs (29).
CD36	472 aa	*O*-glycan (18)	n.d.	In milk, it is involved in protection and nutrition during neonatal development (18). Programming cognitive development (30). Exogenous LCFA transmembrane transport in lactating mammary glands (31).	Mononuclear phagocytes (32). Polysialylation of CD36 in these cells has not been determined.
ESL-1	1179 aa	*O*-glycan (24)	ST8Sia 4 (24)	E-selectin ligand in the Leukocyte adhesion cascade (33).	DCs, monocytes, myeloid cells, and neutrophils (33, 34).
Megalin	4655 aa	*O*-glycan (22)	n.d.	Receptor of apolipoprotein E, Ca^2+^, vitamin B12, polypeptide hormones, and tissue-type plasminogen activator in complex with type-1 inhibitor (22).	n.d.
NCAM	858 aa	*N*-glycan (35, 36)	ST8Sia 4 in immune system (37, 38). ST8Sia 2 and ST8Sia 4 in CNS (39).	Marker for NK cells, high expression in active cytotoxic NK cells (40), mobilization of hematopoietic progenitors (41). Synaptic plasticity, cell adhesion, axon growth and fasciculation in the CNS (42).	DCs, hematopoietic progenitors, microglia, monocytes, neutrophils, NKs (43, 44).
NRP2	931	*O*-glycan (23, 45, 46).	ST8Sia 4 (45, 46).	Receptor for specific isoforms of vascular endothelial growth factors (VEGF) family and for class 3 semaphorins (SEMA3) (47). Angiogenesis (48). Development of selective cranial and sensory nerves, axon guidance, tumorigenesis, vascularization, and cardiovascular development (49–54).	DCs, macrophages and monocytes (23, 43).
Skeletal muscle α-subunit NaV1.4	1836 aa	*N*-glycan (55, 56)	ST8Sia 4 (55, 56)	Generation of action potential in skeletal muscle cells (57).	n.d.
ST8Sia 2	375 aa	*N*-Glycan (58).	ST8Sia 2 (59).	Polysialylation of ST8Sia 2 (autopolysialylation), NCAM in CNS and SynCAM 1 (45).	DCs, hematopoietic precursors, macrophages, monocytes and CD4+ T cells (23, 43, 60).
ST8Sia 3	380 aa	*N*-glycan (13)	ST8Sia 3 (14).	Transfer of polySia and oligoSia to ST8Sia 3 and to NCAM (14, 61). Selective sialylation of several striatum-enriched membrane proteins, adding α2,8-diSia and α2,8-triSia units (62).	n.d.
ST8Sia 4	359 aa	*N*-glycan 16, 63) *O*-glycan	ST8Sia 4 (16, 63)	Transfer of polySia and oligoSia to ST8Sia 4 (autopolysialylation), NCAM, NRP2, ESL-1 and CCR7 (45).	DCs, hematopoietic precursors, macrophages, microglia, monocytes, neutrophils, NK cells, thymocytes, RTEs, CD4+ T cells, CD8+ T cells and B cells (23, 24, 60, 64–67).
SynCAM 1	375 aa	*N-*Glycan (68)	ST8Sia 2 (69)	SynCAM 1 in DCs stimulates IL-22 expression in activated CD8^+^ T-cells (70). In mast cells, SynCAM 1 along with MITF are essential for development and survival of mast cells *in vivo* (71). Involved in Cell adhesion, epithelial integrity and thymus development (70, 72).	Mast cells and DCs (73, 74). Polysialylation of SynCAM 1 in these cells has not been determined.

aa, amino acids; n.d., not determined.

Unlike α2,3- and α2,6-sialylated glycans, which have been easily screened in human cells using lectin panels that use Sia-binding lectins such as *Maackia amurensi*s (MAA II; Siaα2,3) and *Sambucus nigra* (SNA; Siaα2,6) and that have been widely used to determine immune glycophenotypes, no lectins are available to detect polySia (79, 80). Additionally, because of its hydrodynamic arrangement, polySia has been difficult to structurally characterize (81). These challenges have lagged the identification of polySia in other tissues; nonetheless, anti-polySia antibodies with differential specificity for the DP are available, easily allowing the identification of polySia (82). Furthermore, there are now many structural techniques that allow characterization of polySia chains.

In this review, we will summarize the current knowledge on polySia in the immune system, addressing its biosynthesis, its tools for identification and structural characterization, and its functional roles and therapeutic implications.

## Biosynthesis of polySia in Mammals

PolySia is a unique posttranslational modification that consists in linear polymer forms of Sia, joined internally by α2,4, α2,5 *O-glycolyl* α2,8, α2,9, and α2,8/9 linkages (82). In humans, polySia is exclusively formed by the polymeric elongation at position C8 of α2,3- or α2,6-linked Sia, although little is known about the incorporation of dietary Neu5Gc (83).

PolySia was first identified in gram-negative bacterial polysaccharides from pathogens such as *Escherichia coli* K23 and the *Neisseria meningitidis* groups C and B (84, 85). Nonetheless, it is widely expressed in glycoconjugates of the cell surface from bacteria to different types of human cells, although most of its characterization has occurred in CNS tissues (86, 87).

**Figure 1:**
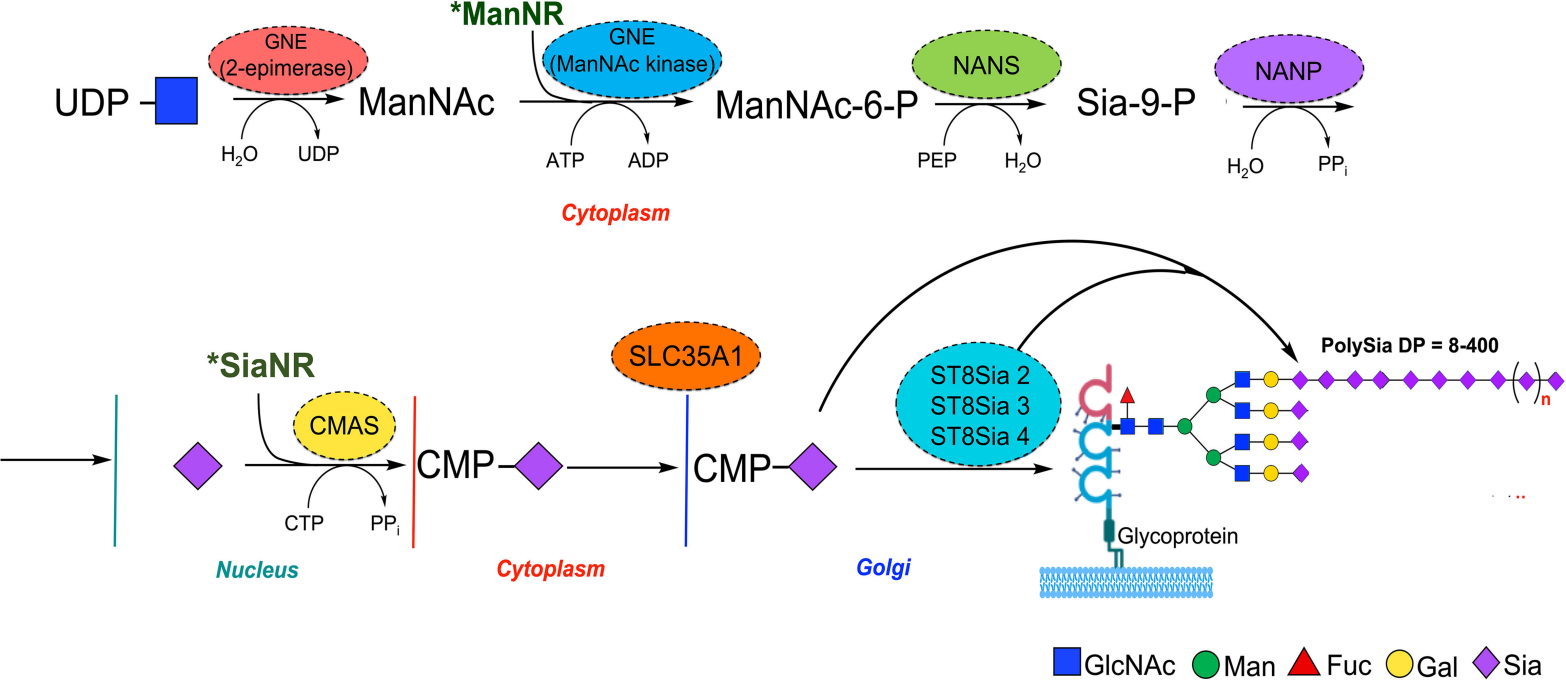
Schematic representation of mammalian polySia biosynthesis. Biosynthesis of PolySia is based on the building block of monomers of Sia through conversion of UDP-GlcNAc into ManNAc by the GNE bifunctional enzyme (epimerase) and subsequently to ManNAc-6-phosphate (kinase). The ManNAc-6-phosphate is then transformed into Sia-9-P by the N-acetylneuraminate synthase (NANS) and dephosphorylated by Sia-9-P-phosphatase (NANP) to yield Sia. After that, Sia is transferred to the nucleus and conjugated with cytidine monophosphate (CMP) by the CMP-Sia synthase (CMAS) and translocated into the Golgi by the CMP-sialic acid transporter SLC35A1, where sialyltransferases, including ST8Sia 2, 3, and 4, use it as donor substrate for polySia synthesis. Synthetic derivatives of ManNAc (ManNR) and Sia (SiaNR) can be used to metabolically label Sia glycans, including polySia.

The biosynthesis of polySia in humans requires the synthesis of CMP-Sia that begins with the assembly of monomeric blocks of Sia through several biosynthetic steps (88, 89) (**Figure 1**). The rate-limiting stage occurs during the conversion of UDP-GlcNAc into *N*-acetylmannosamine-6-phosphate (ManNAc6P) by a single- and dual-UDP-GlcNAc-2 epimerase/ManNAc kinase enzyme (GNE). ManNAc6P is then condensed by the sialic acid synthase (NANS) with phosphoenol-pyruvate resulting in *N*-acetyl-9-phosphoneuraminic acid (Sia9P), followed by dephosphorylation catalyzed by Neu5Ac-9-P phosphatase (NANP). Sia is then translocated into the nucleus where the CMP-Sia synthase (CMAS) activates Sia by transferring the CMP moiety from CTP to the β-anomeric hydroxyl group at C2 of Sia in the presence of Mg^2+^ (90, 91). Unlike all other eukaryotic nucleotide sugar synthetases which are expressed in the cytoplasm, the eukaryotic CMAS enzyme is predominantly located in the nucleus (92). CMP-Sia is then transported to the cytosol by an unknown mechanism, and subsequently, the nucleotide sugar is translocated to the Golgi lumen by the action of the CMP-Sia transporter (SLC35A1) where it is used as a donor substrate by sialyltransferases (STs) for subsequent addition to glycoconjugates (**Figure 1**) (15, 93). In vertebrates, STs are classified into four groups (ST6Gal, ST6GalNAc, ST3Gal, and ST8Sia) according to the glycosidic linkage formed and the sugar acceptor specificity (94, 95). It is important to mention that synthetic derivatives of ManNAc (ManNR) or Sia (SiaNR) can be used to metabolically label sialic acid; examples include ManNAz and SiaNAz where *N*-acetyl is replaced with *N*-azidoacetyl (9).

### The Polysialyltransferases

The ST8Sia enzymes that synthesize the α2,8 Sia linkage, ST8Sia 1 through 6, on glycoproteins or glycolipids (**Table 2** **)**, belong to the CAZy glycosyltransferase family GT29 that show four consensus motifs called sialylmotifs large (SML), small (SMS), motif III (M3), and very small (SMVS) involved in substrate binding, and catalysis (103, 104). In vertebrates, the ST8Sia enzymes have been characterized in fish, mice, and humans (95, 105, 106). Based on sequence analysis, the ST8Sia enzymes can be grouped in monoSTs (ST8Sia 1, 5, and 6) or oligo- and polySTs (ST8Sia 2, 3, and 4) (107). The acceptor substrate specificities and products for the ST8Sia enzymes are shown in **Table 2**.

**Table 2:** Preferred acceptor substrates and products for the human ST8Sia family.

Enzyme	Substrate	Product	Ref.
ST8Sia 1	GM3, GD1a, GT1b gangliosides	GD3, GT1a, and GQ1b gangliosides	(96, 97)
ST8Sia 2	Monosialylated *N*-glycans	DiSia, oligoSia, and polySia (30DP)	(45)
ST8Sia 3	Monosialylated *N*-glycans Keratan sulfate *O*-glycans	DiSia, triSia, oligoSia, and polySia (only autopolysialylation)	(13, 14, 62, 98)
ST8Sia 4	Monosialylated *N*-glycans Oligosialylated *N*-glycans Mucin type *O*-glycans	DiSia, oligoSia, and polySia (50DP)	(39, 46)
ST8Sia 5	GD3, GM1b, GD1a, GT1b, GQ1c	GT3, GD1c, GT1a, GQ1b, GP1c	(99, 100)
ST8Sia 6	α2,3-Sialylated core 1 *O*-glycans	Disia *O*-glycoproteins	(101, 102)

STs from the ST8 family catalyze the transfer of Siaα2,8 to different glycoprotein and glycolipid substrates.

diSia, disialic acid; oligoSia, oligosialic acid; polySia, polysialic acid; triSia, trisialic acid.

The polySTs show a similar structure and are characterized by two motifs likely involved in substrate binding and polySia chain elongation named polysialyltransferase domain (PSTD) of 32 aa located upstream of the SMS, and the polybasic region (PBR) made up of 35 aa that is in the stem region of the enzymes. All the members of the ST8Sia family reside in the Golgi apparatus and possess the catalytic domain tethered to the membrane *via* an N-terminal region and a type II transmembrane domain (107, 108). The amino acid (aa) sequence of the human polyST ST8Sia 4 has 59% identity with that of ST8Sia 2 (109), while the sequence of the human ST8Sia 3 has 33.3% and 34.8% identity with the human ST8Sia 2 and ST8Sia 4, respectively (14).

Regarding NCAM polysialylation in the CNS, it has been observed that both ST8Sia 2 and 4 add polySia to *N*-glycans attached to NCAM more efficiently than to *N*-glycans released from NCAM and that the amount of polySia synthesized by both enzymes is higher than the one obtained by either enzyme alone, exhibiting a synergistic effect (14, 61). Concerning acceptor preferences, ST8Sia 4 is more able with respect to ST8Sia 2 to add polySia to oligosialylated and unpolysialylated antennas in *N*-glycans attached to NCAM, even when polySia is attached to at least one of the other antennas (61). Nonetheless, not all cells express both enzymes and polySia synthesis can be dictated by either ST8Sia 2 or ST8Sia 4. In fact, NCAM polysialylation in immune cells is established by ST8Sia 4 and not by ST8Sia 2 (43).

Autopolysialylation of polySTs is apparently not required to polysialylate NCAM (16, 59, 63, 110). However, ST8Sia 2 and 4 autopolysialylation is required for NRP-2 polysialylation and promotes SynCAM 1 polySia chain elongation (45). Noteworthily, the polyST ST8Sia 3 is also capable of autopolysialylation and presents the PSTD and PBR conserved in ST8Sia 2 and ST8Sia 4 (14, 107, 108). The influence of autopolysialylation in the enzymatic activity of ST8Sia 3 has not been determined.

ST8Sia 3 has not been found to naturally polysialylate NCAM or other known substrates (14, 107, 108). The study of an ST8Sia 3 KO mouse model revealed that ST8Sia 3 is responsible for the selective sialylation of several striatum-enriched membrane proteins, adding α2,8-diSia and α2,8-triSia units to its substrates (62). Nonetheless, these data should be taken cautiously when studying non-neural cells, such as immune cells.

Due to the anti-adhesive properties derived of its large exclusion volume and hydration, polySia can reduce the homophilic or heterophilic interaction in the same membrane (*cis* interaction) or in another cell membrane (*trans* interaction) exhibiting repulsive properties (12). PolySia-repulsive properties are involved in neural cell migration, axonal guidance, fasciculation, myelination, synapse formation, and functional plasticity of the nervous system. In contrast, polySia can also form an attractive field when interacting with soluble molecules such as neurotransmitters, growth factors, and neurotrophic factors directing in many cases binding and release, acting as a reservoir of these molecules on the neural cell surface and as a regulator of the local concentration by condensing them and inhibiting their diffusion (111–113).

It has been shown that polySia binds to brain-derived neurotrophic factor (BDNF), a member of neurotrophin family, forming a complex that allows binding to the BDNF receptor, TrkB, and p75NTR, increasing growth and/or survival of neuroblastoma cells (12). The formation of the BDNF-polySia complex is directly dependent on chain length and requires a DP=12 (12).

Repulsion in polySia-NCAM is differentially regulated by both ST8Sia 2 and ST8Sia 4. Through surface plasmon resonance, it was shown that polySia-NCAM presented different molecule-binding properties depending on the polySTs involved in its synthesis. The polySia-NCAM synthesized by ST8Sia 2 showed a repulsive property toward polySia-NCAM and an attractive field toward BDNF and FGF2 (114). In contrast, polySia-NCAM synthesized by ST8Sia 4 showed only attractive properties toward polySia-NCAM, BDNF, FGF2, and dopamine. This is a consequence of FGF2 and BDNF binding to polySia with DP≥17 and DP≥12, respectively, and as ST8Sia 4 synthesizes larger polySia chains with respect to ST8Sia 2, then polySia synthesized by ST8Sia 4 binds greater amounts of BDNF and FGF2 compared to polySia synthesized by ST8Sia 2 (115). The repulsive field has been observed only in polySia synthesized by ST8Sia 2, but not by ST8Sia 4; however, it is not clear how this homophilic repulsion takes place. This important reservoir function performed by polySia on NCAM has not been explored regarding NCAM-expressing immune cells or other polysialylated proteins.

## Methodologies of PolySia Analysis

The DP of polySia chains has a critical importance in regulating their function. Nonetheless, even with sensitive methods to accurately determine the basic structure, polySia structural characterization, including DP, is still a challenge due to its large size, negative charge, and structural heterogeneity. Some approaches broadly used for polySia analysis may be organized as structural and qualitative, structural and quantitative, quantitative and semiquantitative, or qualitative (**Table 3**) (87, 133).

**Table 3:** Methods for oligoSia/polySia analysis.

Classification	Method	Advantages	Disadvantages	References
**Structural and qualitative analyses**	TLC	Resolves oligoSia from polySia chains. Applied to study the DP of oligoSia composed of different Sia isomers Easy adaptability and inexpensive.	Poor resolution of polySia with greater than 10 Sia units. Requiring at least 1 μg of analyte.	(116)
	MALDI-TOF MS	Determines exact mass composition and DP of polySia. Preferential detection of unmodified peptides and partial or complete suppression of glycopeptides. Low quantities (ng) of polysialylated proteins can be analyzed. Discerns between α2,9 linked polySia from α2,8 linked polySia. PolySia of DP up to 100, and 40 Sia units have been successfully detected.	Poor tolerance to sample impurities. Remotion of peptides and enrichment of glycopeptides is needed. The mass accuracy and the resolution of the signals are reduced in the linear mode and did not allow an identification of incompletely lactonized species.	(117)
	ESI-MS	Determines linkages and structure. Low quantities from 10 to 20 pmol of the compound with Sia moieties can be analyzed. Sia dimers, trimers, and tetramers can be detected with higher efficiency.	Derivatization approach is important to analyze sialylated glycans without losing terminal sia groups.	(118, 119)
**Structural and quantitative analyses**	HPAEC-PAD	Allows detection of all non-volatile and most semi-volatile analytes. It is not necessary to derivatize samples. PolySia can be quantified by coupling HPAEC with a detector based on amperometry, fluorescence, UV absorbance, or mass spectrometry.	To detect DP 50 is necessary 10 μg of purified polySia samples. Epimerization and degradation of carbohydrates. Unstable baseline, loss of sensitivity, and requirement of a dedicated base compatible HPLC.	(120, 121)
	HPAEC-FD	Widely employed method. High sensitivity. Can detect polySia with DP > 90 Amount of 200 ng derivatized colominic acid have been analyzed. Can also detect polySia from tissues with DP ranging from 18 to 60	Derivatization process with DMB requires acidic conditions and longer periods of incubation. Cationic charges getting for derivatized polySia are critical for separation.	(122)
	HPAEC-UV	Quantify-free polySia with a resolution of up to 25 Sia units. Does not require derivatization.	Poor selectivity using short UV wavelength (210 nm) by increasing background. High sample purity is necessary. Among 10 μg purified polySia for analysis	(123, 124)
	HPAEC-CAD	CAD separates polySia of higher degree of polymerization >90 colominic acid units. Can detect DP among 65 and 130 Sia residues. Does not require derivatization. Less time than other methods. Less specific and less sensitive than HPAED-FD. Lower amount (250 ng)	Large amount of polySia polymer is necessary. Restricted to volatile buffers Decreased resolution by increased salt. High standards of sample purity, compared to fluorometric detection.	(125–127)
**Quantitative analysis**	ELISA	Reproducible and reliable method. High specificity and sensitivity. Can analyze very small samples. Rapid and accurate for quantitation of total polysialylated proteins.	The method cannot distinguish polySia of different chain lengths.	(128)
	Flow cytometry	Can detect polySia on the surface of intact cells. The anti-PolySia antibodies and Endo N-GFP fusion proteins can be used in flow cytometry allowing the analysis of the number of polySia positive cells. It is selective and sensible.	To corroborate PolySia antibody specificity, sometimes it is necessary to use the Endo N enzyme. Specificity is associated with antibodies and controls must be used. The exact DP is not possible to determine.	(129, 130)
	Fluorometric C7/C9	Highly sensitive and selective analysis of internal Sia residues of oligo- and polySia. Internal Sia residues that remain unaffected can be analyzed by HPLC-FD after fluorescence derivatization. Detection of 1-ng amounts of internal Sia residues of oligo- and polySia molecules.	False positive quantitative results for internal Sia residues of polySia. The method allows oxidation of α2,9 linked polySia.	(131, 132)
**Semiquantitative or qualitative analysis**	Western blotting	Expression levels of polysialylated-proteins. Associated chemiluminescent and/or fluorescent signals. High specificity. Detects polySia residues with 735 antibody and 12E3 antibody recognizes oligo/polySia.	Less accurate to quantify polysialylated-proteins. Smeared bands. The quantity of polySia determined is lower than with the ELISA method.	(58)

TLC, thin layer chromatography; MALDI-TOF MS, matrix-assisted laser desorption/ionization mass spectrometry; ESI-MS, electrospray ionisation mass spectrometry; HPAEC, high-performance anion exchange chromatography; PAD, pulsed amperometric detection; FD, fluorometric detection; UV, ultraviolet detection; CAD, charged aerosol detector; ELISA, enzyme-linked immunosorbent assay.

The structural characterization of polySia requires in most cases a combinational analysis or high-throughput analysis techniques (133). One of the first analytical approaches used for polySia analysis was thin-layer chromatography (TLC) of mild acid hydrolysate of polySia, using resorcinol as a visualization reagent (116, 124, 134, 135). Nowadays, structural and qualitative analyses such as MALDI-TOF and ESI-MS offer many advantages over traditional analytical methods, including low sample consumption and high sensitivity (117, 135).

Furthermore, the structural and quantitative analyses of polySia consists of several steps such as mild acid hydrolysis, derivatization, and HPLC analysis of DMB-polySia derivatives that allow determination of polySia DP and glycosidic linkages. One of the most suitable and recommended approaches to polySia analysis is high-performance anion-exchange chromatography with pulsed amperometry detector (HPAEC-PAD) (136). The major advantage of HPAEC-PAD is that this approach requires no derivatization for sensitive detection and allows the inclusion of different detectors offering diverse ways to detect polySia such as fluorescence (HPAEC-FD), ultraviolet (HPAEC-UV), and corona charged aerosol detection (HPAEC-CAD). HPAEC analysis can characterize poly/oligosaccharides using a pellicular anion-exchange resin and sodium hydroxide phase (87). The Neu5Ac and Neu5Gc residues are stable in alkaline eluents, but sialic acids containing *O*-acyl substituents are unstable, then a gradient of sodium acetate is recommended. Within quantitative methods, periodate fluorometric C7/C9 analysis is available to detect inter Sia residues of oligo and polySia. It consists of periodate oxidation of Sia residues and fluorescent labeling with DMB detecting α2,8-linked oligo/polySia structures using combinatorial platforms such as reverse-phase HPLC with fluorescence detection (RPLC-FD) (116, 117, 136, 137). An additional approach that provides similar results and uses soft acid conditions is mild acid hydrolysis–fluorometric anion-exchange chromatography method (MH-FAEC), recently adapted for oligo-PolySia analysis (138).

The semiquantitative or qualitative analysis represented by Western blot (WB) and quantitative analysis such as ELISA and flow cytometry are mostly based in immunodetection of polySia (138, 139). A disadvantage of ELISA is that it cannot distinguish polySia of different chain lengths and WB only offers relative quantification of polySia based on differential densitometry associated with chemiluminescent and fluorescent signal obtained from blots. Unfortunately, it is frequent in WB to visualize polySia as wide and smeared bands that difficult quantification. Flow cytometry is a rapid high-throughput approach that allows detection and measurement of polySia expression in cells by detecting fluorescence intensity using anti-polySia antibodies conjugated with fluorophores or fusion proteins such as GFP-tagged engineered endoneuraminidase enzyme (EndoN-GFP) that has been modified to bind but not digest polySia (140). Advantages and disadvantages for several methodologies to analyze polySia are described in **Table 3**.

The anti-di/oligo/polySia antibodies can be classified into three groups based on their specificity for chain DP (44, 82) (**Table 4**). The group I antibodies are the “anti-polySia antibodies” that recognize chains of α2,8-linked Sia with ≥DP 8, including fully extended polySia chains. These antibodies recognize the helical conformation formed by Sia residues within the internal region of the polySia chains. The non-reducing terminal residues are not involved in antigen recognition. The group II antibodies recognize both oligoSia with DP = 2–7 and polySia chains. These antibodies recognize the distal portion of oligo/polySia chains, including the non-reducing terminal. The group III antibodies recognize oligoSia with DP = 2–4, but do not bind to polySia. These antibodies appear to recognize specific conformations of di/oligoSia with DP = 2–4. The combinatory use of antibodies allows estimation of polySia DP. The two most used anti-polySia antibodies are the monoclonal 12E3 antibody (IgM) that recognizes the non-reducing terminal residue of oligoSia/polySia acid structures with DP≥5 and the monoclonal 735 antibody (IgG) that recognizes the internal sialyl residues of polySia structures with DP≥11.

**Table 4:** Anti-oligoSia/polySia antibodies.

Group	Clone	Organism	Immunoglobulin type	Immunogen	Type of sialic acid recognized	DP specificity
**I) Anti-polySia**	H.46	Horse	poly, IgM	*Neisseria meningitidis GpB*	Neu5Ac	DP≥8
	735	Mouse	Mono, IgG2a	*Neisseria meningitidis GpB*	Neu5Ac	DP≥11
**II) Anti-oligoSia + anti-polySia antibody**	12E3	Mouse	Mono, IgM	Embryonic rat forebrain	Neu5Ac	DP≥5
	5A5	Mouse	Mono, IgM	Membrane from embryonic rat spinal cord	Neu5Ac	DP≥3
	2-2B	Mouse	Mono, IgM	*Neisseria meningitidis GpB*	Neu5Ac	DP≥4
	OL.28	Mouse	Mono, IgM	Oligodendrocyte from newborn rat	Neu5Ac	DP≥4
	2-4B	Mouse	Mono, IgM	Oligo/polyNeu5Gc-PE	Neu5Gc	DP≥2
	Kdn8kdn	Mouse	Mono, IgM	KDN-gp	KDN	DP≥2
**III) Anti-oligoSia antibody**	S2-566	Mouse	Mono, IgM	Human GD3	Neu5Ac	DP=2
	1E6	Mouse	Mono, IgM	(Neu5Ac)2-bearing artificial glycopolymer	Neu5Ac	DP=2
	AC1	Mouse	Mono, IgG3	(Neu5Gc)GD1c	Neu5Gc	DP=2–4
**Other**	12F8	Rat	Mono, IgM	Mouse membrane fraction	Unknown	Unknown

Poly, polyclonal; mono, monoclonal.

Finally, the use of neuraminidases is a helpful resource to identify the presence or composition of Sia in cells. EndoN is a phage enzyme that specifically degrades α2,8 Sia polymers, diffusing rapidly in tissues and capable of degrading polySia in cultured cells (141). EndoN is highly specific for polySia and requires a minimum DP of 5 to act. Higher DP 150–200 are better substrates for EndoN than oligomers DP 10–20 (142). Treatment with EndoN of purified or whole lysates is indicated to confirm anti-polySia antibody specificity (23). The EndoN-treated proteins can be analyzed by WB to confirm a weight shift related to polySia; alternatively, the liberated glycan product can be subjected to structural analysis. EndoN can also be incubated in cell cultures *in vitro* to evaluate the functional roles of polySia, as well as real-time fluorescence microscopy (143) and flow cytometry (144). Injected EndoN has also been used *in vivo* to evaluate the role of polySia (145).

## PolySia in the InnateImmune Response

The innate immune response is the body’s first line of defense against pathogens entering the body. The innate immune response uses different mechanisms to stop the spread of infections such as mucous membranes, physical barriers, defense cells, and proteins (4). Sia are essential epitopes which have an important role in self-recognition and regulation of immune system cells (7, 146). The polySia mediates cell–cell interactions and promotes signaling through steric and electrostatic exclusion, making polysialylated glycans key participants in migration and inflammation (8, 82). Additionally, polySia is considered as a recognition pattern and immune regulator in the immune innate response (7). Further research is required to fully elucidate the receptors through which polySia acts, including the known interactions with DC-SIGN and Siglecs. In this regard, the identification of Siglecs as receptors for sialic acid–containing glycans, including polySia, is quite interesting in view of their expression on most white blood cells and their critical role in immune cell signaling, as well as to distinguish between self and non-self (147). PolySia is also known to bind the myristoylated-alanine-rich C-kinase substrate (MARCKS) expressed in neurons to modulate neuritogenesis (148); interestingly, although MARCKS is also expressed in neutrophils and macrophages, its interaction with polySia in these cells has not been reported (149, 150).

### Bone Marrow Hematopoiesis

**Figure 2:**
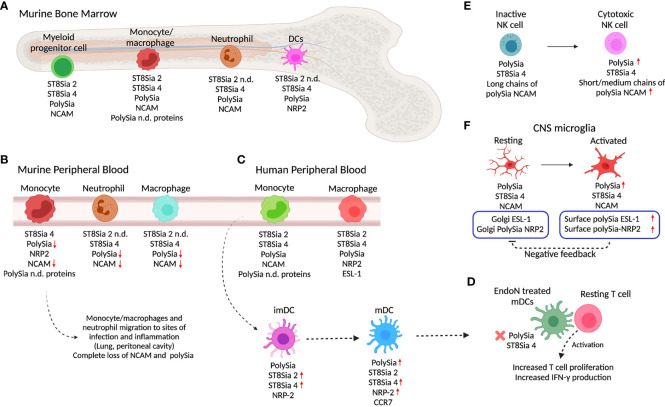
PolySia and polyST expression in innate immune cells. **(A)** In the murine bone marrow (BM), different myeloid progenitor cells, monocytes/macrophages, neutrophils, and DC express polySia, ST8Sia 2, ST8Sia 4, NRP2, NCAM, or non-identified polySia proteins. **(B)** As they migrate into the peripheral blood, murine BM-derived monocytes/macrophages and neutrophils lose polySia-NCAM. As the macrophages and neutrophils arrive at infection or inflammation sites, polySia is depleted from the cell surface. **(C)** In the periphery, human macrophages and monocyte-derived cells express polySia and polySia carriers such as NRP2, NCAM, ESL-1, and unknown proteins. Once monocytes differentiate into imDC, they express polySia-NRP2 which in turn is upregulated during maturation to mDC. **(D)** Depletion of polySia by using EndoN neuraminidase in mDCs promotes increased T cell activation. **(E)** NK cells overexpress polySia and ST8Sia 4 during differentiation into cytotoxic cells. Chain length differentiates between non-cytotoxic and cytotoxic NK cells. **(D)** The depletion of polySia in mDC by using EndoN induces better activation and increased proliferation of T cells, suggesting a role of polySia in regulation of T cell activation. **(F)** In the innate immunity of the CNS, microglia cells also express polySia. The increase in polySia-NRP2 expression in the surface of microglia cells induces negative feedback on Golgi polySia-NRP2 localization and expression. The increase or decrease of polySia expression with respect to the precursor state is indicated with red arrows (up or down, respectively). Created with BioRender.com.

PolySia has been detected in murine bone marrow (BM) cells and myeloid precursors, using anti-polySia monoclonal antibody (mAb) 735 and a green fluorescent protein (GFP)-fused and inactive endoglycosidase N (EndoN-GFP), which binds specifically to but does not cleave α2,8 polySia. PolySia was detected on the surface of >60% of cells aspirated from the murine BM, with ST8Sia 2 and ST8Sia 4 gene expression being detected in BM cells (43). Nonetheless, ST8Sia 4 was shown to be the one responsible for polySia synthesis of BM resident populations and during myeloid differentiation as was observed when the expression of polySia was conserved in BM cells from ST8Sia 2^-/-^ mice and absent in cells from ST8Sia 4^-/-^ mice (43, 130) (**Figure 2A**). Further analysis of activity and expression of ST8Sia 2 might help to explain the mechanism that leads to ST8Sia 4 as responsible for polySia synthesis in BM cells.

Murine BM polySia expression has been correlated with receptor tyrosine kinase c-kit (hematopoietic progenitor marker) and defines four BM subsets from a common myeloid lineage: polySia^neg^/Kit^high^, polySia^low^/Kit^high^, polySia^high^/Kit^high^, polySia^low^/Kit^low^). The polySia^neg^/Kit^high^ subset contained hematopoietic stem cells (c-Kit^+^, Sca-1^+^), the polySia^low^/Kit^high^ population, committed multipotent progenitors (CD34^+^), and the polySia^high^/Kit^high^, polySia^low^/Kit^low^ groups, immature, and mature myeloid cells, respectively (130). In contrast to murine BM cells, human fetal BM cells, myeloid cells, and peripheral myeloid precursors cells do not express polySia. Differences between murine and fetal human BM cells were attributed to the intrinsic polySia expression variability during development (130).

Also, polySia has been detected in murine BM neutrophils and monocytes/macrophages that express the myeloid lineage markers CD11b (Mac-1) and/or Ly6 G/C (Gr-1). PolySia was not detected on the surface of lymphocytes or erythroid cells. PolySia was found to be controlled by expression levels of ST8Sia 4 and associated with cell surface NCAM, as well as to other unknown polysialylated proteins in certain subpopulations of murine BM monocytes (43).

During migration of murine BM cells, polySia is shed from the surface of monocytes by cleavage of NCAM as they are released from BM into the PB during early stages of monocyte differentiation and completely lost when they localize in pulmonary and peritoneal sites of inflammation (43) (**Figure 2A**). This is probably due to metalloprotease-induced ectodomain shedding that has been described in neurons (151). Additionally, in ST8Sia 4^-/-^ mice lacking polySia-NCAM that is required for the mobilization of hematopoietic progenitors from the BM to the thymus with lack of polySia causes retention in the BM and improper access to the thymus for maturation (41).

Differently to murine BM monocytes/macrophages where ST8Sia 2 and ST8Sia 4 are expressed, murine BM neutrophils express only the ST8Sia 4 associated with the biosynthesis of polySia-NCAM which is also progressively lost by protein cleavage as they migrate from the BM to the PB and completely lost when they localize in pulmonary and peritoneal sites of inflammation (**Figures 2A, B** **)** (43). It has been proposed that the regulation of polySia in monocyte populations might be associated with discrimination of cells on functional grounds or lineage commitment; however, the loss of polySia could also allow migration by increasing the overall negative charge, reducing cell–cell contact, and regulating the differentiation and maturation of different cell subsets.

### Dendritic Cells

The dendritic cells (DCs) present antigen to naive T cells at specific intercellular junctions called immunological synapses (152). Monocytes are precursors of peripheral non-lymphoid organ DCs and migratory DCs under inflammatory conditions (153). Maturation of DCs is associated with reprogramming of the glycosylation machinery, especially sialylation (154).

NRP2 is a protein expressed by murine and human DCs and known to be polysialylated (23, 43) (**Figures 2A, C** **)**. Human peripheral monocytes express both polySTs but do not express polySia-NRP2. The *de novo* NRP2 protein expression and polysialylation occur during differentiation into immature DCs (imDCs) mediated by IL-4 and GM-CSF. The monocyte-derived imDCs upregulate ST8Sia 2 and ST8Sia 4 polyST and increase polySia expression (**Figures 2A, C** **)** (23). After LPS stimulation to induce maturation of human imDCs into mDCs, ST8Sia 4 is highly overexpressed while ST8Sia 2 remains at the same level as imDCs. In human mDCs, polySia-NRP2 is exclusively polysialylated on *O*-glycans by ST8Sia 4 (23, 46). Similarly, in murine monocytes, the expression of polySia-NRP2 is regulated during stages of differentiation/activation. As occurs in human monocytes, murine BM-derived monocytes do not express polySia-NRP2 that only occurs during their migration from BM to PB (43)(**Figures 2A, B** **)**. Interestingly, like human mDCs, mouse BM-derived DCs express ST8Sia 4 and polySia-NRP2 (23).

The TLR4 stimulation of monocyte-derived imDCs causes the upregulation of ST8Sia 4 and polySia expression in immunogenic mDCs, similarly to the effect caused by IL-4 and GM-CSF. Prolonged TLR-4 engagement though LPS stimulation is required for the generation of polySia-expressing human monocyte-derived mDCs, which is also required for the CCL21 capture and subsequent CCL21-mediated migration (155). However, unlike monocyte-derived mDCs, monocytes, and monocyte-derived immature mDCs do not overexpress polySia or ST8Sia 4 after prolonged (2 days) LPS stimulation (155). Also, the TLR4 stimulation of human monocyte-derived tolerogenic mDCs does not cause polySia overexpression, although polySia and ST8Sia 4 are expressed by these cells (155).

In NRP2^-/-^ mice, it has been observed that BM monocyte-derived DCs also express polySia in NCAM, as well as other unidentified carrier proteins (**Figure 2A**). Unlike NRP2, which is upregulated during migration from BM to PB, polySia-NCAM is downregulated in BM-derived monocytes (**Figure 2B**) (43).

The blocking of polySia in NRP2 with anti-polySia IgG or digestion with EndoN to remove polySia from the cell surface of DCs enhances their ability to activate T cells, suggesting that polySia-NRP2 regulates the activation of T cells by DCs (23) (**Figure 2D**). Also, the digestion with EndoN showed that stimulation with CCL21 and phosphorylation of Akt and JNK kinases are reduced when polySia is removed, indicating that polySia is required for signaling of CCL21 through CCR7. The depletion of ST8Sia 4 by knockdown in DCs resulted in the reduction of CCL21-mediated migration (156). Similarly, it has been reported that CCR7 and CCL21 contribute to the migratory capacity of DCs within the skin and to the lymph nodes (155).

PolySia also influences migration of imDCs located in the periphery where they capture pathogens and migrate as mDCs to draining lymph nodes to activate T cells. Migration of mDCs to lymph nodes was abrogated in ST8Sia 4^-/-^ mice (25).

CCR7 is the central chemokine receptor controlling immune cell trafficking to secondary lymphatic organs. There are data that support that CCR7 is polysialylated in murine BM-derived mDCs and that this modification is essential for CCL21 ligand recognition (25). HEK293 cells transfected to co-express CCR7-GFP fusion protein and ST8Sia 4 showed polySia-CCR7, and mutational analysis demonstrated that both *N*- and *O*- glycans are associated with CCR7 polysialylation. Moreover, flow cytometry by using the anti-polySia 735 monoclonal antibody of murine BM-derived CCR7^-/-^ mDCs showed reduced polysialylation in comparison to control DCs, suggesting that additionally to NRP2, CCR7 is also polysialylated (25). EndoN digestion experiments in human mDCs could be conducted to evaluate if CCR7 is also polysialylated.

Noteworthily, SynCAM 1 (Necl2/CADM1), a member of the immunoglobulin superfamily of transmembrane glycoproteins, mostly known as a neural synaptic adhesion molecule with multiple functions, is also expressed in a specialized subset of murine and human DCs, where it was shown to interact through its extracellular domain with CRTAM (class I MHC-restricted T cell-associated molecule), a receptor expressed in cytotoxic lymphocytes to preserve epithelial integrity and required for proper thymus development (157, 158). SynCAM 1 in DCs was also found to regulate IL-22 expression by activated CD8^+^ T cells (70, 72). SynCAM 1 has also been found to be expressed by mast cells where it is involved in driving mast cell-sensory neuron adhesion and promoting the development of a microenvironment in which neurons enhance mast cell responsiveness to antigen (73). SynCAM 1 on tumors has also been shown to interact with CRTAM, promoting cytotoxicity of NK cells and interferon-gamma secretion by CD8^+^ T cells *in vitro* as well as NK cell-mediated rejection of SynCAM 1-expressing tumors (159). Also, it has been shown that epithelial to mesenchymal transition (EMT) induced SynCAM 1 expression, regulating NK-mediated, metastasis-specific immunosurveillance in balance with E-cadherin (160). However, despite these important functional observations, the polysialylation of SynCAM 1 has not been evaluated in DCs or in the context of interactions with other types of immune cells.

### NK Cells

The NCAM protein is used as a marker for NK cells and their subpopulations; however, the function of polySia-NCAM on NK cells is still not clear. Mature human NK cells are divided into 2 subsets based on the relative surface density of the NCAM antigen: NCAM^pos^ cells, predominantly found in secondary lymphoid tissues, and NCAM^neg^ cells, predominant in PB (161) (**Figure 2E**). Differently to the observations in human cells, adult murine NK cells do not express ST8Sia 4 or polySia, with polySia and NCAM expression being restricted to multipotent hematopoietic progenitors and cells derived from the myeloid lineage (130). However, fetal mouse BM-derived NK cells do express polySia, although polyST expression has not been evaluated (130).

In human peripheral NK cells, polySia biosynthesis was found driven by ST8Sia 4 as no expression of ST8Sia 2 was detected (37, 38). The short- (DP 1-10) or medium- (DP 11-140) length polySia chains on NCAM are characteristic of active cytotoxic NK cells, while larger chains (DP 141-370 +) are expressed by cytotoxic inactive NK cells (**Figure 2E**) (130).

Weakly polysialylated NCAM in NK cells binds in trans-homophilic interaction with DC-SIGN and plays a very important role in the fate of DCs. This interaction inhibits homotypic intercellular interactions of NCAM^pos^ cells and protects DC-SIGN-expressing DCs against NCAM^pos^ cell-mediated cytotoxicity (40).

PolySia-NCAM in NK cells also interact with polySia-NCAM expressed by tumor cells. The CRISPR-Cas9 deletion of NCAM in the NK cell line NK-92 showed a reduction in killing of NCAM^+^ tumor cells (162). Also, the loss of NCAM protein reduced cytotoxicity and lytic granule exocytosis (163). These data strongly support that polySia and NCAM are implicated in the regulation of NK cell cytotoxicity.

Recently, the expression of ST8Sia 4 and polySia expression were evaluated in human peripheral NK cells and different NK cell lines under the activation of IL-2. Upon activation, there was no change in ST8Sia 4 expression; however, polySia expression increased, and this was explained by increased NCAM expression (38, 162) (**Figure 2E**). Despite the relevance of polySia in the function of NCAM in other cells, the specific function of polySia in the NCAM of NK cells has not been evaluated.

### Microglia and CNS Immune Response

Under homeostatic conditions, the adaptive immune response of the CNS is very limited, and the innate immune response depends on endogenous brain cells, in particular the microglia. Microglial cells play a pivotal role in brain development, maturation, and homeostasis by responding to infection, trauma, or other pathological conditions, transforming into macrophage-like cells with a professional innate immune defense function that can be regulated by sialylation (164–166). Sialic acids maintain the homeostasis of the CNS innate immune response by inhibiting complement, including microglia *via* Siglecs and other receptors (167).

Polysialylated NCAM has been studied mainly in the development of the nervous system, particularly in neuronal processes such as migration, cytokine response, and differentiation dependent on cell contact (168). Due to these characteristics, it is not surprising that polysialylated molecules also participate in immunological processes (130).

Several studies have used the NCAM^-/-^ mice as a model to evaluate the role of polySia-NCAM in different functions such as neuronal connectivity, plasticity, and migration (169). In the microglia context, the role of polySia in NCAM^-/-^ mice has also been evaluated (170). The NCAM interaction modulates the activation of microglia, and it is responsible for homophilic binding in microglial immune response through production of nitric oxide (NO) and TNFα (171, 172).

Residual polySia signals in the brain of NCAM^-/-^ mice indicated the presence of alternative polySia carriers (173). In murine NCAM^-/-^ microglial cells, polySia was found to be carried by both NRP2 and ESL-1 and synthetized by ST8Sia 4 (**Figure 2F**). In the case of NRP2, it has been identified to be present in *O*-glycans. Golgi-localized polySia-NRP2 and polySia-ESL-1 appeared during injury-induced activation of murine microglia, and inflammatory activation by stimulation with LPS caused their translocation to the cell surface with subsequent depletion by ectodomain shedding (24, 143). The same mechanism was found in the differentiation of human THP-1 monocytic cells into macrophages, where polySia in NCAM disappears, but polySia-ESL-1 and polySia-NRP2 are detected in the Golgi and depleted upon proinflammatory activation with LPS, but not through anti-inflammatory activation induced by IL-4 **(** **Figure 2F** **)** (24).

In a model of traumatic brain injury, Golgi retention of polySia proteins was found abrogated by calcium depletion of the Golgi compartment that induced the translocation and rapid depletion of polySia from the Golgi to the cell surface (66). Additionally, depletion of the microglia cell surface polySia occurred through ectodomain shedding induced by metalloproteinase activity, although other reports indicate that it is mediated by exovesicular Neu1 neuraminidase (175). The polySia degradation was shown to cause liberation of the neurotrophic factor BDNF that polySia binds (175). The liberated BDNF from polySia chains would supply this and other neurotrophic factors to injured tissue.

Also, soluble polySia has been proposed to participate in the negative feedback regulation of proinflammatory microglia activation as it attenuates NO production of LPS-induced stem cell-derived microglia and reduced TNFα and IL-1β mRNA levels. LPS-induced NO production of NCAM-deficient microglia increased by the additional deletion of ST8Sia 4, that is, by the inability to produce polysialylated NRP2 or ESL-1 (24). This negative feedback has been proposed to occur through human Siglec-11 interaction or glutamate receptor function (170, 176). In the case of mouse microglia, this had not been observed in view that the inhibiting Siglec-11 has no mouse orthologue. Nonetheless, it was recently shown that this effect was mediated by *trans* interaction with murine Siglec-E, a mouse orthologue of human Siglec-9, expressed in the myeloid lineage (177). CRISPR/Cas9-mediated Siglec-E knockout led to a strong LPS response and failed in preventing inhibition of proinflammatory activation by exogenous polySia (66).

According to a study on Parkinson’s disease, the administration of intraperitoneal polySia in humanized Siglec-11 transgenic mice showed neuroprotective properties after repeated injections with LPS, indicating that polySia is a potential drug candidate for preventing Parkinson’s disease-associated inflammation and neurodegeneration. Brain transcriptome analysis showed increased levels of immune-related genes that prevent exacerbated immune responses as well as the loss of dopaminergic neurons in the substantia nigra *pars compacta* induced by LPS (178).

Taken together, these data show that polySia has a potential anti-inflammatory function in brain microglia, particularly through interaction with the Siglec-11 receptor, regulating the signaling inflammatory responses and retaining the microglial homeostasis. Nonetheless, these results must be taken cautiously as they are derived from mice that present a more restricted expression of Siglec members, 9 compared to 14 in humans. Specifically, the humanized Siglec-11 transgenic mice do not co-express the human Siglec-16, the paired activating receptor of Siglec-11.

Upon engagement of Sia-containing ligands, inhibitory Siglecs such as Siglec-11 recruit cytoplasmic tyrosine phosphatases SHP1/2 to their ITIM domain to deliver inhibitory signal(s) that modulate and counteract immune responses. Therefore, the available data point out to polySia using Siglecs to establish a negative feedback regulation of the immune response. The human Siglecs known to bind polySia include the inhibitory Siglec-9 and Siglec-11 (179, 180). Other inhibitory Siglecs known to recognize α2,8 Sia glycans in the form of disialic acid include Siglec-5 and Siglec-7, although there are no data regarding their binding of polySia (181).

It is thought that because certain pathogens developed mimicry to evade the immune response by engaging inhibitory Siglecs, Siglecs with activating signaling potential evolved, such as human Siglecs-14 and 16, in which the ITIM/ITIM-like intracellular domains are replaced with an immunoreceptor tyrosine-based activation motif that recruits the activating adapter protein DAP12 (147). Certain inhibiting and activating Siglecs function as paired receptors and are typically expressed together; such is the case of Siglec-5 and Siglec-14 and Siglec-11 and Siglec-16 (182). It is important to note that although no polySia binding has been identified in activating Siglecs, Siglec-14 and 16 are known to recognize α2,8 Sia glycans in the form of disialic acid (183, 184).

Therefore, the role of therapeutic polySia and its signaling through Siglecs must be further analyzed in the context of the inhibiting and activating responses that occur in human physiology, particularly considering that these responses, compared to murine Siglecs, are very probably more complex.

### Neutrophils and Macrophages

Neutrophils are polymorphonuclear and phagocytic leukocytes of the innate response that act against pathogens; they are also important effector cells during tissue injury-induced inflammation (185). Murine BM-derived neutrophils have surface expression of polySia-NCAM associated with ST8Sia 4 activity (43). Like murine monocytes, during the exit and migration from BM, neutrophils lose the expression of polySia-NCAM until its complete depletion once they reach the alveolar and peritoneal sites of inflammation (43) (**Figures 2A, B** **)**. The relevance of polySia-NCAM depletion during migration has not been evaluated; however, loss of polySia could have a similar effect to the loss observed when Sia was cleaved by sialidase treatment, improving adhesion and migration of neutrophils (65).

As occurs with the BM-derived monocytes and neutrophils, BM-derived macrophages decrease polySia surface expression during migration. Neutrophils and macrophages lose polySia when they migrate from the BM into the PB and then to pulmonary and peritoneal sites of infection or inflammation (43). When peritoneal macrophages were induced *in vitro* to repolysialylate by inducing a more quiescent state of activation, polySia was re-expressed and found to be carried by NRP2 and other unidentified proteins. Also, EndoN treatment for polySia removal from monocytes, as they mature into macrophages *in vivo* during recruitment to inflammatory sites, improved phagocytic activity of the *Klebsiella pneumoniae* pathogen, indicating that progressive loss of polySia during migration to inflammation sites is necessary for efficient phagocytosis (43).

Murine peritoneal macrophages express CD36 and Siglec-E. In murine macrophages, CD36 is a highly glycosylated protein that mediates the modified low-density lipoprotein (LDL) uptake (186). Both CD36 and Siglec-E interact during oxidized LDL (oxLDL) uptake in macrophages (187). The interaction leads to downregulation of CD36 signaling regulating in the uptake of oxLDL, which in turn promotes foam cell formation that participates in atherogenesis (188). Sialidase treatment showed that Sia is not required for Siglec-E and CD36 interaction but is required for CD36 SHP-1/VAV signaling involved in LDL uptake, through unknown membrane components (187). The CD36 protein is also found in mammalian milk where it is reported to be polysialylated; however, although CD36 in macrophages was found to be predominantly modified by α2,6-linked sialylation, polysialylation has not been evaluated (18, 189).

In the context of infection, the ST8Sia 4-coding gene was found upregulated in human monocyte-derived M2-like-polarized macrophages infected with human rhinovirus (RV), a single-stranded RNA virus, that causes asthma exacerbation (190). The role of the increased ST8Sia 4 or the presence of polySia has not been investigated during RV infection; however, the authors suggest the potential role of the upregulated genes in the polarization to the M2-like phenotype enhancing the RV-induced type 2 cytokine expression (190).

As mentioned in previous sections, polySia has been found to be a negative feedback regulator of the immune response, a finding with therapeutic implications for diseases such as Alzheimer’s disease. The polySia with an average DP of 20 (polySia avDP 20) promoted anti-inflammatory functions in human THP-1 macrophages through its interaction with Siglec-11, inhibiting the LPS-induced gene transcription and protein secretion of TNFSF2 and preventing the oxidative burst associated with phagocytosis of Alzheimer’s disease-associated fibrillary amyloid-β_1–42_. In addition, polySia avDP20 neutralized the LPS-triggered increase in macrophage phagocytosis, showing that polySia DP is relevant for determining the biological effect (191).

Also, the development of polySia-coated nanoparticles demonstrated that polySia binds to histones in NET fibers. The release of histones may be intentionally triggered to the cell surface during apoptosis or to the extracellular fluid during NETosis, a regulated form of neutrophil cell death that contributes to the host defense against pathogens through formation of neutrophil extracellular traps (NETs), which consist of modified chromatin decorated with bactericidal proteins (192, 193). Extracellular histones have cytotoxic properties because they are procoagulant and proinflammatory and are also toxic for mammalian epithelia and endothelia, contributing to the microvascular dysfunction observed in sepsis and autoimmune diseases (194, 195).

PolySia would have a role in protecting endogenous cells against histone-mediated cytotoxicity on the basis that application of polySia decreases the bactericidal function of histones (196). The polySia chains of secreted NCAM were shown to neutralize the cytotoxic activity of extracellular histones as well as DNA/histone-network-containing “neutrophil extracellular traps,” which are formed during invasion of microorganisms. The interaction of polySia with histones appears to be improved with increased DP, showing better binding with DP 24–32 and 32–38 in comparison to shorter DP; also, both DP 24–32 and 32–38 improve the migration to distance of histones through binding to polySia, which could impact the cytotoxic function of histones. Similarly, low polySia of DP 15–24 does not influence the migration but does participate in cytotoxicity (197).

NETs are also loaded with lactoferrin that forms a shell around neutrophils, suppressing the release of NETs. Recent evidence suggests that polySia binds not only to histones but also to lactoferrin of NETs and that the expression of polySia regulates the accumulation of external lactoferrin, regulating the formation of NETs by neutrophils (198).

The regeneration of tissue after application of exogenous polySia has been previously studied in the CNS for potential therapeutic use, evaluating the role in axonal growth of polySia (199). Polysia is upregulated in the murine CNS during transplantation of polySia-overexpressing Schwann cells, improving regeneration after spinal cord injury (200). The intravitreal application of polySia with avDP 20 in a macular degeneration laser-damage murine model reduced mononuclear-phagocyte activation and tissue damage as well. PolySia avDP 20 prevented membrane attack complex (MAC) deposition in wild-type and in humanized Siglec-11 transgenic mice. *In vitro*, polySia inhibited the reactivity of mononuclear phagocytes, preventing TNF-α, VEGF-A, and superoxide production, but also *via* Siglec-11 receptors interfering with activation of the alternative complement system and preventing the phagocytosis-associated oxidative burst (201). Siglecs expressed by mononuclear phagocytes recognize Sia as a self-associated molecular pattern (SAMP) functioning as sensors for “self” (202). As mentioned previously, these results derived from humanized transgenic mice must be taken cautiously as they do not include the co-expression of Siglec-16, the paired activating receptor of Siglec-11.

## 5 Polysialic Acid Role in the Adaptive Immune Response

The adaptive immune system takes over if the innate response is not able to clear and destroy pathogens. The adaptive response acts through cytokine mechanisms where effector, cytotoxic, plasmatic, and regulatory cells orchestrate the response. Sia is involved in the regulation of B and T cell maturation, differentiation, migration, and cell survival or cell death fate (203, 204). The presence of polySia has only been characterized in T cells, but not B cells.

Sia is actively involved in pathogen recognition through interaction with glycan-binding proteins and in regulating key pathophysiological steps within T cell biology such as T cell development and thymocyte selection, T cell activity and signaling, and T cell differentiation and proliferation (205). These roles highlight the importance of Sia as a determinant of either self-tolerance or T cell hyperresponsiveness which ultimately might be implicated in the creation of tolerogenic pathways in cancer or loss of immunological tolerance in autoimmunity (202, 203).

Although both ST8Sia 2 and ST8Sia 4 are expressed in BM hematopoietic precursors (and in both primary and secondary human lymphoid organs), the expression and regulation of polySia have been poorly investigated in association with the adaptive immune response. ST8Sia 2 expression has been identified in the adult human thymus, while ST8Sia 4 is abundantly expressed in primary and secondary lymphoid organs such as the placenta, spleen, thymus, intestine, and the PB (206).

**Figure 3:**
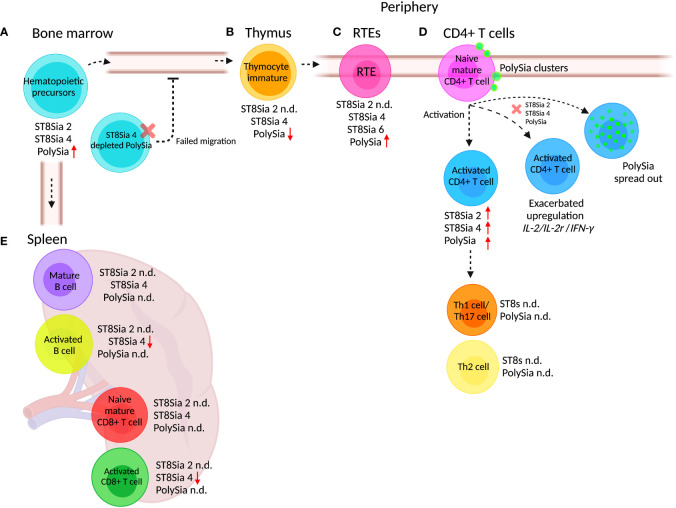
PolySia and polySTs expressed in adaptive immune cells. During differentiation of hematopoietic precursors, maturation, migration, and activation, the cells of the adaptive response suffer changes in the expression of polySia and the polySTs. **(A)** Hematopoietic precursors derived from BM express ST8Sia 4 and ST8Sia 2, but the polySia is synthesized exclusively by ST8Sia 4. In ST8Sia 4^-/-^ mice, the BM hematopoietic progenitors fail to exit, migrate, and access the thymus for maturation into T cells. **(B)** Hematopoietic progenitors mature in the thymus where they differentiate into immature thymocytes which in turn express polySia synthesized by ST8Sia 4. **(C)** After maturation in the thymus, recent thymic emigrants (RTEs) migrate through the circulation to reach the lymph nodes. The RTEs express the ST8Sia 4 and ST8Sia 6 as well as polySia. **(D)** Peripheral naive CD4^+^ T cells express ST8Sia 2, ST8Sia 4, and polySia, which are overexpressed upon *in vitro* stimulation. When ST8Sia 2 and ST8Sia 4 are downregulated by knockdown (red x) in naive CD4^+^ T cells, the upregulation of *IL-2*, *IL-2r* (IL-2 receptor), and *IFNγ* genes is exacerbated during activation. **(E)** Activation of mature B cells reduces ST8Sia 4 expression, but polySia expression has not been analyzed. Naive and activated CD8^+^ T cells express ST8Sia 4; however, such as occurs with B cells, the polySia expression has not been determined (n.d). Different types of lymphocytes express ST8Sia 4, but polySia expression has not been determined (n.d.). The increase or decrease in polySia expression with respect to the precursor state is indicated with red arrows (up or down). Created with BioRender.com.

The T cell progenitors are produced in the BM and mobilized to the periphery at regular intervals by signals to reach the thymus where they mature. The ST8Sia 4^-/-^ mice show a reduction in total thymocytes and a concomitant deficiency in the earliest thymocyte precursors in comparison to multipotent hematopoietic progenitors derived from wild-type ST8Sia 4 mice with normal polySia synthesis (41). *In vivo* reconstitution of polySia expression in ST8Sia 4^-/-^ hematopoietic progenitors showed that defective T cell development is caused by improper access to the thymus (**Figure 3A**) (41). These results suggest that the observed defect in thymocyte development is not due to abnormalities in T cell development, but is related to the inability of these polySia-negative cells to exit the BM and travel to the thymus (41). Because of its increased size, steric hindrance, and negative charges, polySia has been identified as an anti-adhesive molecule (207), modulating the distance between cell–cell interaction of cell–epithelia heterotypical or homotypical interaction. This can explain why BM cells that do not express polySia in ST8Sia 4^-/-^ mice are unable to modulate the interaction in the BM niche, creating an inefficient exit of BM cells and migration to the thymus.

During maturation in the thymus, T cells suffer phenotypic and functional changes derived from thymic environment interaction (208). The interaction of the thymocyte with different surrounding cells determines the fate of their differentiation and maturation, as well as the exit from the thymus. PolySia is a key regulator of cell–cell contact, and as is mentioned above, ST8Sia 4 is responsible for polySia synthesis in hematopoietic precursors. According to the immunological genome project database, ST8Sia 4 is downregulated during the development and maturation of thymocytes, in agreement with observations where polySia is upregulated during differentiation and maturation to generate egress from the BM, requiring downregulation to promote thymic retention (67, 209).

Newly generated peripheral T cells designated as recent thymic emigrants (RTEs) continue post-thymic maturation in secondary lymphoid organs to become long-lived naive T cells (210). The increase in Sia expression on the cell surface glycans after maturation is a signature of thymocyte maturation (**Figure 3B**). Cre-NKAP^-/-^ mice (NKAP is a transcriptional repressor that binds to histone deacetylase 3 required at several points in hematopoiesis) fail to complete T cell maturation because RTEs are eliminated by the complement system (211). Defective α2,8 sialylation occurs in NKAP^-/-^ T murine cells because of downregulation of ST8Sia 1, ST8Sia 4, and ST8Sia 6 (**Figure 3C**). Apparently, α2,8 sialylation is critical in RTEs to avoid complement fixation and removal (211).

After maturation, naive T cells need to be activated in the secondary lymphoid organs such as lymph nodes, spleen, and Peyer’s patches. During activation, T cells must receive two signals, firstly from an antigen-presenting cell (APC) *via* the immunological synapse, initiated by the TCR recognition of an antigen peptide displayed on the MHC of an APC (212), and secondly from a costimulatory binding mediated by CD28 on T cells and CD80/CD86 ligands on the APC (213). In this context, T cell activation is regulated by the concentration and affinity of antigen, duration of antigen stimulation, and the costimulatory signals and the cytokine environment present at the time of antigen presentation, as well as glycosylation changes, including sialylation (214).

The expression of Sia has been studied in different cell subsets of peripheral T cells and B cells. Previous observations of Sia changes during activation of human peripheral and murine splenocytes showed α2,3 and α2,6 Sia hyposialylation, because of the downregulation of ST3 and ST6 STs (67, 215, 216). This hyposialylation on both CD4^+^ T and CD8^+^ T cells has been associated with induction of apoptosis to regulate the homeostasis of these cell populations (217). It is important to remark that murine resting CD8^+^ T cells express ST8Sia 4 which is downregulated after activation; however, the expression of polySia has not been evaluated in these cells (67).

The hyposialylation of α2,3 and α2,6 Sia linkages during activation of peripheral human naive CD4^+^ T cells was found to be accompanied by global cell surface sialylation at the expenses of α2,8 Sia (60). During activation of CD4^+^ T cells, ST8Sia 2 and ST8Sia 4 were found to be upregulated and no expression of ST8Sia 3 was observed (60). The upregulation of ST8Sia 2 and ST8Sia 4 upon activation increased polySia expression in a subpopulation of human peripheral naive CD4^+^ T cells (**Figure 3D**) (144). In mice, the activation of CD4^+^ T, CD8^+^ T cells, and B cells induces the downregulation of ST8Sia 4; however, the expression of polySia has not been evaluated (67).

The expression of polySia in human CD4^+^ T cells was associated with different unknown protein carriers. Additionally, the cell surface expression of polySia in resting naïve CD4^+^ T cells occurred in a clustered pattern, which dispersed after activation (144). The knockdown of both ST8Sia 2 and ST8Sia 4 in human resting naïve CD4^+^ T cells exacerbated the expected overexpression of *IL-2*, *IL-2R*, and *IFN-γ* genes after anti-CD3/CD28 activation, indicating that polySia glycoconjugates participate in the negative regulation of the activation response of human peripheral CD4^+^ T cells (**Figure 3D**) (144). Differences occur between these observations and reports in murine splenocytes where activation of CD4^+^ T and CD8^+^ T cells with anti-CD3 plus IL-2 or of B cells with anti-IgM plus IL-4 causes downregulation of ST8Sia 4 (67)(**Figure 3E**). Furthermore, the presence of polySia in murine T cells or B cells has not been reported, limiting the comparison between these two models.

The expression of NCAM has been detected in γδ T cells. Although these cells represent less than 5% of all T cells, they act as a first line of defense in the skin, gut, and reproductive tract while other lymphocytes are still being developed, performing distinct roles in pathogen clearance, wound healing, autoimmunity, and cancer, supporting the functions of DC, T cells, and NK lymphocytes through both innate and adaptive properties including antigen-presenting capabilities (218). The proportion of NCAM^+^ γδ T cells appears to be determined by their level of activation. NCAM^+^ γδ effector T cells produce large amounts of IFN-γ after stimulation and are more resistant to apoptosis. Additionally, NCAM expression is stronger in proliferating cells and gradually disappears with the number of cell divisions. Thus, NCAM expression is considered to define the γδ T cells with the highest antitumor activity (219). The antibody-mediated blocking of NCAM or removal of polySia chains in NCAM^+^ γδ T cells reduced cell proliferation and caused lower lytic effector activity (220, 221), although further investigation is required to understand the role of polySia in the antitumoral function of NCAM^+^ γδ T cells.

## Discussion

Although most of what we know about the biological roles of polySia has originated from its study in the CNS, and particularly from NCAM, this review shows that different types of cells of the immune system express polySia in NCAM but also in different protein carriers, known and unknown. The accumulated evidence in both innate and adaptive immune cells reveals that polySia is a key regulator of immune cell biology, from hematopoiesis to effector functions, something that is not unexpected from a polymer with such potent biophysical characteristics and that has already been characterized to be dynamically modulated during CNS development.

The use of anti-polySia antibodies has proven very valuable to approach the identification of polySia in cells; nonetheless, it is also important to advance toward a more specific structural characterization of polySia, particularly DP, as length chain also determines the biological information encoded by this glycan polymer. Most of the studies included in this review address the expression of polySia using specific antibodies, but a limited number have determined the DP composition of polySia. It is important to consider that as DP is linked to ST8Sia 2 and/or ST8Sia 4 activity, it would also be subject to dynamic regulation, resulting in heterogeneity at different stages of cellular differentiation and activation that could be part of an additional fine-tuning of the immune response, including differences at the level of the human population. It is therefore important to apply combinatorial techniques for a more precise structural analysis of polySia. Additionally, although ST8Sia 3 has polySia synthesis activity that is apparently limited to autopolysialylation, it is important to evaluate its expression and potential role on polySia synthesis along with the ST8Sia 2 and ST8Sia 4, particularly among the diversity of immune cell types where the role of this enzyme has not been thoroughly studied.

Although the functional relevance of polySia has been clearly demonstrated in all the immune cell types where it has been found, more is needed to identify its *cis*- and *trans*- ligands. The interaction of polySia with members of the Siglec family in different immune cells is very promising to identify signaling pathways as has been shown for microglial cells and macrophages that act through interaction with Siglec-11, inhibiting microglial activation, inflammation, phagocytosis, and oxidative burst (146). Additionally, the identification of the signaling pathways of polySia, particularly involving inhibiting Siglecs, also paves the way to developing polySia-based therapeutics, particularly through its anti-inflammatory properties. However, further research needs to address the net effect of paired inhibiting and activating Siglec receptors to truly model the complexity of Siglec signaling.

Although polySia has been mostly characterized in the cell surface, studies in microglia indicate that intracellular polySia dynamics associated with retention and release is a mechanism linked to changes in ionic concentrations (Ca^2+^, Mn^2+^, and Mg^2+^) that influence polySTs activity. This is very important in understanding polySia dynamics in immune cells where ionic concentration also fluctuate and are of key importance during activation (118, 222). It is therefore critical to address both cell surface and intracellular polySia expression to fully comprehend the involved regulatory mechanisms. This can also be extended to phosphorylation mediated by protein kinase C that regulates signal transduction pathways important for both innate and adaptive immunity and that is known to positively regulate the activity of both ST8Sia 2 and ST8Sia 4 in the polysialylation of murine NCAM in the CNS (223).

A systematic exploration in all human immune cells of the expression and functional role of polySia, as well as its carriers and ligands, promises to reveal potent mechanisms through which this glycan polymer acts throughout the different stages of immune cells. There is a critical mass of information available to extrapolate the different findings on the role of polySia in different types of immune cells, from both mice and humans, to readily accelerate this field of research.

## Author Contributions

TV-C and IM-D designed this review. All authors contributed equally to the literature revision and manuscript writing. All authors contributed to the article and approved the submitted version.

## Funding

IM-D and TV-C were supported by grant RT-279765 from Red Temática Glicociencia en Salud - Consejo Nacional de Ciencia y Tecnología (CONACyT) 2019 and Ciencia Básica-CONACyT grant 282454 and the Sociedad Latinoamericana de Glicobiología, A.C. LG-V was supported with a scholarship from CONACyT 882333.

## Conflict of Interest

The authors declare that the research was conducted in the absence of any commercial or financial relationships that could be construed as a potential conflict of interest.

## Publisher’s Note

All claims expressed in this article are solely those of the authors and do not necessarily represent those of their affiliated organizations, or those of the publisher, the editors and the reviewers. Any product that may be evaluated in this article, or claim that may be made by its manufacturer, is not guaranteed or endorsed by the publisher.
